# Detection of Spatiotemporal Prescription Opioid Hot Spots With Network Scan Statistics: Multistate Analysis

**DOI:** 10.2196/12110

**Published:** 2019-06-17

**Authors:** Arinjoy Basak, Jose Cadena, Achla Marathe, Anil Vullikanti

**Affiliations:** 1 Biocomplexity Institute of Virginia Tech Blacksburg, VA United States; 2 Lawrence Livermore National Laboratory Livermore, CA United States; 3 Network Systems Science and Advanced Computing Division Biocomplexity Institute and Initiative University of Virginia Charlottesville, VA United States; 4 Department of Public Health Sciences University of Virginia Charlottesville, VA United States; 5 Department of Computer Science University of Virginia Charlottesville, VA United States

**Keywords:** opiate dependence, spatial temporal analysis, network scan statistics, medical specialty

## Abstract

**Background:**

Overuse and misuse of prescription opioids have become signiﬁcant public health burdens in the United States. About 11.5 million people are estimated to have misused prescription opioids for nonmedical purposes in 2016. This has led to a signiﬁcant number of drug overdose deaths in the United States. Previous studies have examined spatiotemporal clusters of opioid misuse, but they have been restricted to circular shaped regions.

**Objective:**

The goal of this study was to identify spatiotemporal hot spots of opioid users and opioid prescription claims using Medicare data.

**Methods:**

We examined spatiotemporal clusters with signiﬁcantly higher number of beneﬁciaries and rate of prescriptions for opioids using Medicare payment data from the Centers for Medicare & Medicaid Services. We used network scan statistics to detect signiﬁcant clusters with arbitrary shapes, the Kulldorff scan statistic to examine the signiﬁcant clusters for each year (2013, 2014, and 2015) and an expectation-based version to examine the signiﬁcant clusters relative to past years. Regression analysis was used to characterize the demographics of the counties that are a part of any signiﬁcant cluster, and data mining techniques were used to discover the specialties of the anomalous providers.

**Results:**

We examined anomalous spatial clusters with respect to opioid prescription claims and beneﬁciary counts and found some common patterns across states: the counties in the most anomalous clusters were fairly stable in 2014 and 2015, but they have shrunk from 2013. In Virginia, a higher percentage of African Americans in a county lower the odds of the county being anomalous in terms of opioid beneficiary counts to about 0.96 in 2015. For opioid prescription claim counts, the odds were 0.92. This pattern was consistent across the 3 states and across the 3 years. A higher number of people in the county with access to Medicaid increased the odds of the county being in the anomalous cluster to 1.16 in both types of counts in Virginia. A higher number of people with access to direct purchase of insurance plans decreased the odds of a county being in an anomalous cluster to 0.85. The expectation-based scan statistic, which captures change over time, revealed different clusters than the Kulldorff statistic. Providers with an unusually high number of opioid beneficiaries and opioid claims include specialties such as physician’s assistant, nurse practitioner, and family practice.

**Conclusions:**

Our analysis of the Medicare claims data provides characteristics of the counties and provider specialties that have higher odds of being anomalous. The empirical analysis identifies highly refined spatial hot spots that are likely to encounter prescription opioid misuse and overdose. The methodology is generic and can be applied to monitor providers and their prescription behaviors in regions that are at a high risk of abuse.

## Introduction

### Background

Overuse and misuse of prescription opioids have become signiﬁcant public health burdens in the United States [1]. More than one thirds of the US civilian population is reported to have used prescription opioids in 2016, as per estimates from the National Survey on Drug Use and Health (NSDUH) [2]. One third of Medicare Part D beneficiaries received prescription opioids in 2017 [3]. Furthermore, about 11.5 million people are estimated to have used prescription opioids for nonmedical purposes in 2016 [1,2]. Pharmaceutical opioids are now among the most popular drugs for nonmedical use in the United States, second only to marijuana [4]. This has led to problems of drug overdose and deaths. According to the Centers for Disease Control and Prevention (CDC), there were more than 63,600 drug overdose deaths in the United States in 2016 [5], and there were over 70,000 deaths in 2017.

One of the major factors driving the opioid crisis is the misuse of prescription opioids; about 40.6% of people reported obtaining them from family or friends [1]. According to the International Narcotics Control Board, in 2009, the United States consumed 99% of the world’s hydrocodone, 60% of the world’s hydromorphone, and 81% of the world’s oxycodone. The number of prescriptions written for opioids increased by 300% between 1991 and 2009 [6], and research by Wisneiwski et al (2008) [7] shows high correlations among admissions to addiction treatment facilities, overdose deaths, and the volume of opioids prescribed in the United States.

Public and private health insurance plans have been criticized for lacking consistency in treating chronic pain conditions and in providing viable alternative medical treatments. For example, an extensive study by Lin et al (2018) [8], which considers 15 Medicaid plans, 15 Medicare Advantage plans, and 20 commercial insurers in 2017, which covers half the US population, finds that their health insurance policies offer inconsistent policy terms for nondrug treatments for chronic lower back pain, and they provide little or no coverage for alternative treatments that have scientific backing.

It has also been shown from NSDUH reports that there are signiﬁcant racial and demographic patterns associated with opioid misuse, for example, white males and lower socioeconomic characteristics [9]. These ﬁndings motivate a careful spatiotemporal analysis of prescription rates for opioids to (1) discover regions with unusually high opioid prescription rates and (2) understand what distinct factors in these regions could be associated with higher usage.

To this end, spatial scan statistics [10-12] are among the most common techniques used for analyzing metrics related with opioid use. Linton et al [13] analyze spatial patterns of emergency and nonemergency calls (911 and 311) related to narcotics in Baltimore, Maryland. They identify clusters of calls in some neighborhoods, coinciding with urban redevelopment. Brownstein et al [14] use patient data from New Mexico at the level of zip codes to identify locations of clusters. They characterize the structure and locations of signiﬁcant clusters within the state. Cordes [4] analyzes mortality because of prescription opioids, using the North Carolina State Center for Health Statistics’ Injury Free North Carolina database.

There has also been some analysis of prescription data at different spatial resolutions. CDC reports prescription rates for different states and demographics [15]. Prescription rates at a county level are reported using the QuintilesIMS Transactional Data Warehouse data [16]. However, these reports only provide visualizations of the spatial data, but they do not identify clusters that are different from the rest of the state or that might have changed over time.

### Objectives

This study aimed to fill this gap by identifying spatiotemporal hot spots of opioid users and opioid prescription claims using Medicare Part D data. It applies network scan statistics to detect signiﬁcant clusters with arbitrary shapes and an expectation-based version to examine the signiﬁcant clusters relative to past years. Even though this analysis is focused on Virginia, West Virginia, and North Carolina, the methodology is generic and can be applied to other regions and in other contexts for anomaly detection. Its application to spatial epidemiology can help detect unimmunized or underimmunized clusters of individuals in an otherwise well-vaccinated population [17]. Other possible application areas are detection of suicide clusters, teenage pregnancies, and criminal activity [18,19].

Once the clusters are identified, the census data from those spatial regions are analyzed to characterize the demographic attributes of the counties that are a part of the anomalous clusters.

## Methods

### Ethical Considerations

This study did not require Institutional Review Board approval, as only publicly available, open source, data were used.

### Datasets

#### Centers for Medicare & Medicaid Services Data

We used the Medicare Provider Utilization and Payment Data: Part D Prescriber Public Use File (PUF), made available by the Centers for Medicare & Medicaid Services (CMS) [20]. The Part D Prescriber PUF contains data on all prescription drugs prescribed by individual physicians and other health care providers and paid for under the Medicare Part D Prescription Drug Program. Each provider is identified by a National Provider Identifier, along with the provider’s name and address, which are used to map them to counties in the United States. For each provider, the dataset gives information on the number of beneficiaries seen and the number of prescriptions dispensed at the provider’s direction. The dataset also separately lists the number of prescriptions for opioids and the corresponding number of beneficiaries for such prescriptions. These data are available for 2013, 2014, and 2015.

#### Demographic Data

We used American Community Survey (ACS) census datasets from the US Census Bureau website [21] to analyze the demographic properties of clusters discovered by network scan statistics. Statistics on race, sex, origin, and number of housing units in the counties were covered by the ACS datasets PEPANNHU and PEPSR6H, respectively. Economic characteristics of the population were covered by the DP03 dataset, which gives information on employment status by type of employment, health insurance coverage, subtypes of income characteristics of the population, and some other economic variables. Income distribution for each county were obtained from the S1901 dataset. Table B27019 in ACS provides health insurance coverage characteristics by 2 age groups, that is, ages 25 to 64 and 65 and above; it further breaks down each group by educational attainment. B27002-3 and C27004-7 datasets provide health insurance coverage by age groups, sex, and the kind of health insurance, that is, (1) public or private, (2) Medicare or Medicaid, and (3) in case of private health insurance, whether the plan is employer supported or directly purchased from a private company or exchange. Each one of these datasets is available at different geographic resolutions, starting with the county-level data. Data are available for the years 2013, 2014, and 2015. Different combinations of variables were extracted from these datasets, and the details of the variables used are also mentioned in the *Characterization of Demographics in Clusters* section.

### Detecting Clusters Using Network Scan Statistics

Spatial scan statistics are some of the most commonly used approaches for detecting anomalous clusters from spatial data [10-12], and they have been applied in a number of public health problems, including detection of opioid activity and related metrics [4,13,14,22].

We briefly described the Poisson version of the Kulldorff scan statistic [10], which we use in our analysis. Let ζ denote the set of counties in the state. For each county i∈ζ and time t (which, in our case, is the year), we consider 2 kinds of counts: (1) tot_b_(i,t) and tot_p_(i,t), which are the total number of beneficiaries and prescriptions, respectively, in county i—these are the *baseline* counts for time t—(2) opioid_b_(i,t) and opioid_p_(i,t), which are the total number of beneficiaries and prescriptions for any kind of opioid drug, respectively, for county i—these are the *event* counts for the county at time t. For a cluster C⊂ζ, we define tot_p_(C,t) = ∑_i∈C_ tot_p_(i,t) and opioid_p_(C,t) = ∑_i∈C_ opioid_p_(i,t). Similarly, tot_b_(C,t) and opioid_b_(C,t) are defined. We will drop the dependence on time t from the notation, when it is clear from the context.

The Kulldorff scan statistic is based on hypothesis testing. The null hypothesis H_0_ is that the event counts for all counties i are proportional to their baseline counts:

H_0_: opioid_p_(i) ~ Poisson(µ·tot_p_(i))

Where µ is the statewide rate for opioid prescriptions: opioid_p_(ζ)/tot_p_(ζ). Under the alternative hypothesis H_1_(C) for a cluster C⊂ζ, the prescription event counts for counties within C are generated proportionally to some rate η, whereas event counts for counties outside C are generated with some rate µ<η:

H_1_(C) = opioid_p_(i) ~ Poisson(η·tot_p_(i)), i∈C;
opioid_p_(i) ~ Poisson(µ·tot_p_(i)), i∉C

The Kulldorff scan statistic F(C) is defined as a generalized likelihood ratio,

F(C) = Pr[data|H_1_(C)]/Pr[data|H_0_(C)]

and the objective is to find clusters C with maximum F(C) value, as these clusters are likely to have a significantly higher rate than the statewide average.

One of the popular tools for finding such clusters is the SaTScan software (Martin Kulldorff, SaTScan) [23], which considers clusters with circular or elliptical shapes and returns those with the maximum statistic score.

We also searched for clusters where the number of beneficiaries or prescriptions have changed significantly from 1 year to the next. Due to the temporal aspect of this task, we considered the expectation-based Poisson Scan Statistic [24], which compares event counts observed at the present and the past. Under the null hypothesis, the event counts in year t come from the same model as the counts in the year t–1 for each county: opioid_p_(i,t)~Poisson(opioid_p_(i,t–1)). Under the alternative, there is a cluster C⊂ζ, where the counts are generated at a higher rate than expected: opioid_p_(i,t)~Poisson(µ·opioid_p_(i,t–1)), for some µ>1.

For both functions, statistical significance of the clusters was assessed via Monte Carlo sampling. We followed the approach from the study by Kulldorff [10] and generated replicates of the data under the null hypothesis, conditioned on the total event counts. The score of any cluster in the original data is compared with the distribution of the optimal solutions on the replicates to get its *P* value. For example, a *P* value of .05 indicates that the F(C) score of the cluster we report is in the top 5% of scores obtained in the null replicates; we used 1000 replicates in our experiments.

### Network Scan Statistics

We used an extension of scan statistics that is applied to networks. We defined a graph G=(ζ,E) on the set of counties, where 2 counties i,j∈ζ are connected by an edge (i,j)∈E if they share a boundary. We focused on clusters C⊂ζ that induce a connected subgraph in G—this allows us to consider clusters that might be arbitrarily shaped, instead of being restricted to disks or ellipses. We searched for a connected cluster that maximizes the Kulldorff scan statistic. Computationally, this turns out to be much harder, and we used the approach developed in the study by Cadena et al [25] for optimizing different kinds of scan statistics on networks. This method provably gives clusters that are connected subgraphs, with a bounded size, with the maximum likelihood score. We say that a cluster is *significant* if it has a *P* value of .05, as computed using the Monte Carlo method described above. We first find clusters with the highest score F(C), computed using the method in the study by Cadena et al [25], and then consider them only if they are also significant.

### Regression Method for Characterizing Counties Contained in Opioid Clusters

We performed a logistic regression analysis to identify demographic factors that could help characterize the composition of counties that are identified as anomalous. Separate regressions were run for each of the years, for each of the 3 states, and for both types of clusters, that is, clusters with higher than normal opioid-beneficiary counts and opioid-prescription claim counts.

In each case, the response variable is constructed from the network scan statistics–based clusters that are found to be anomalous in terms of counts. All counties in the state were labeled 1 if they belonged to an anomalous cluster; otherwise, they were labeled 0. The independent variables were extracted from the US Census data for each state. We started with 30 independent variables that included information on race, income, access to private, public, and employer-based insurance, direct purchase insurance, access to Medicare and Medicaid, education level, number of housing units, number of disabled, employment rate, different levels of income-to-poverty ratios, and distribution of male and females in the county.

We measured cross correlations among these variables and removed variables that had 60% or higher correlation among them. If multiple variables are correlated, only one of them is selected. Removing correlated variables addresses the problem of multicollinearity, which results in standard errors of the coefficients to be large [26-28]. After removing all the correlated variables, only 8 independent variables remained.

These 8 variables are the following: (1) percentage of African Americans in the county, (2) percentage of American Indians in the county, (3) percentage of males in the county, (4) number of housing units in the county, (5) percentage of people with access to Medicaid, (6) percentage of people with access to Medicare, (7) percentage of access to direct purchase insurance, and (8) percentage of households with income-to-poverty ratio less than 0.5. Income-to-poverty ratio measures a family’s income with respect to the poverty threshold. If it is less than 1, it implies that the household income is below poverty line. Variable (8) represents the percentage of households in the county that have income-to-poverty ratio less than 0.5; it represents the households who live in extreme poverty.

The regression analysis started with these 8 variables. We use the *stepAIC* function given in R software (The R Foundation) to apply the forward selection and backward elimination method to find the most relevant subset of predictors for the regression [29,30]. This method finds the model that minimizes the information loss, as measured by the Akaike Information Criterion (AIC). The stepAIC function begins with a full or null model, and method for stepwise regression can be specified in the direction argument with values *forward*, *backward*, or *both*. Our method used *both*. For each state, each year, and for each type of counts, we separately trained the model. In each case, a subset of the original 8 independent variables was selected, on the basis of the AIC. The selected variables in each case are shown in the tables with regression results. If a variable is missing altogether, it implies it was not selected in the final model in any of the years for the state. If it is used in only some of the 3 years, the coefficient is shown as ‘-’ in the years the variable is not selected. For a variable to be considered significant, the *P* value should be less than .1.

## Results

### Overview

The empirical analysis has been performed on 3 states, namely Virginia, North Carolina, and West Virginia. The results for the state of Virginia are described in detail, but for West Virginia and North Carolina, only a brief summary is provided to avoid repetitive discussion. Interested readers can find detailed results on West Virginia and North Carolina in the Multimedia Appendix 1.

### Significant Clusters in Virginia

#### Location and Characteristics Based on Opioid Beneficiary Counts

**Figure 1:**
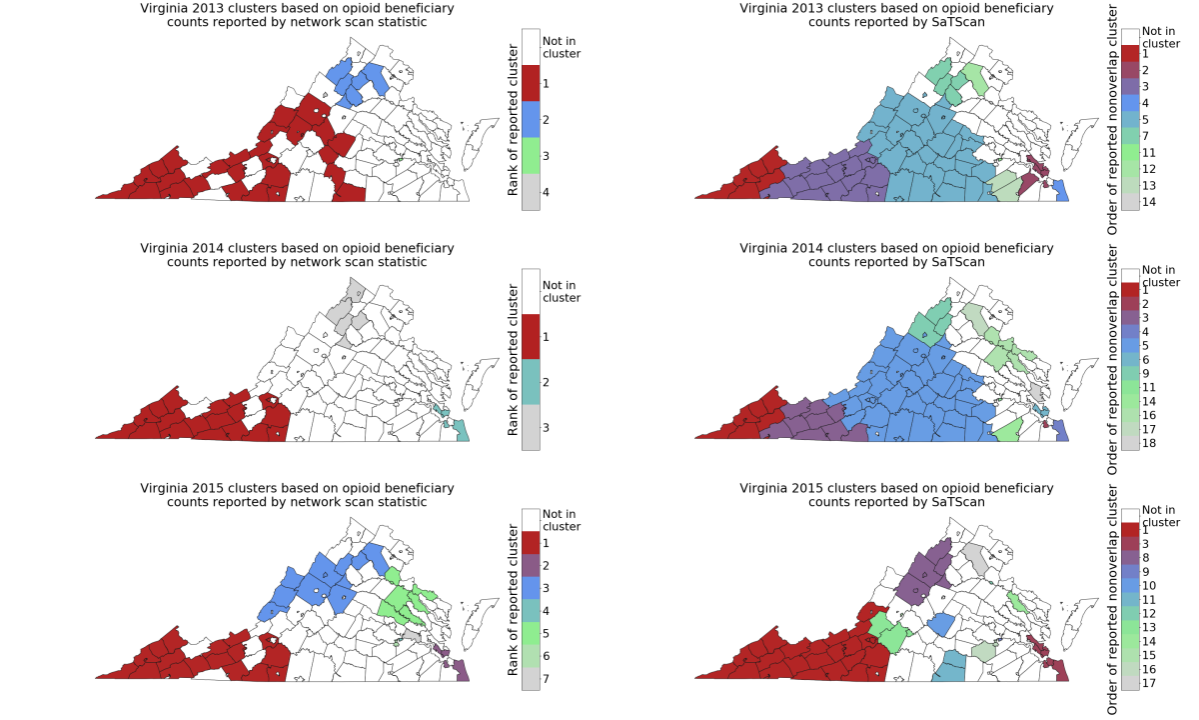
Clusters discovered in Virginia for 2013 (top), 2014 (middle), and 2015 (bottom), using network-based approach (left) and SaTScan (Martin Kulldorff, SaTScan; right) on opioid beneficiary counts.

Figure 1 shows the clusters for different years, discovered using the network-based approach (left) and SaTScan (Martin Kulldorff, SaTScan; right). The clusters are ranked in nonincreasing order of scan statistic score, F(C), mentioned earlier. We observed changes in the number and composition of clusters over time: there is a large cluster of 30 counties in southwest Virginia in 2013, which shrank in 2014 and 2015. On the other hand, significant clusters in other parts of the state emerged in 2015.

**Figure 2:**
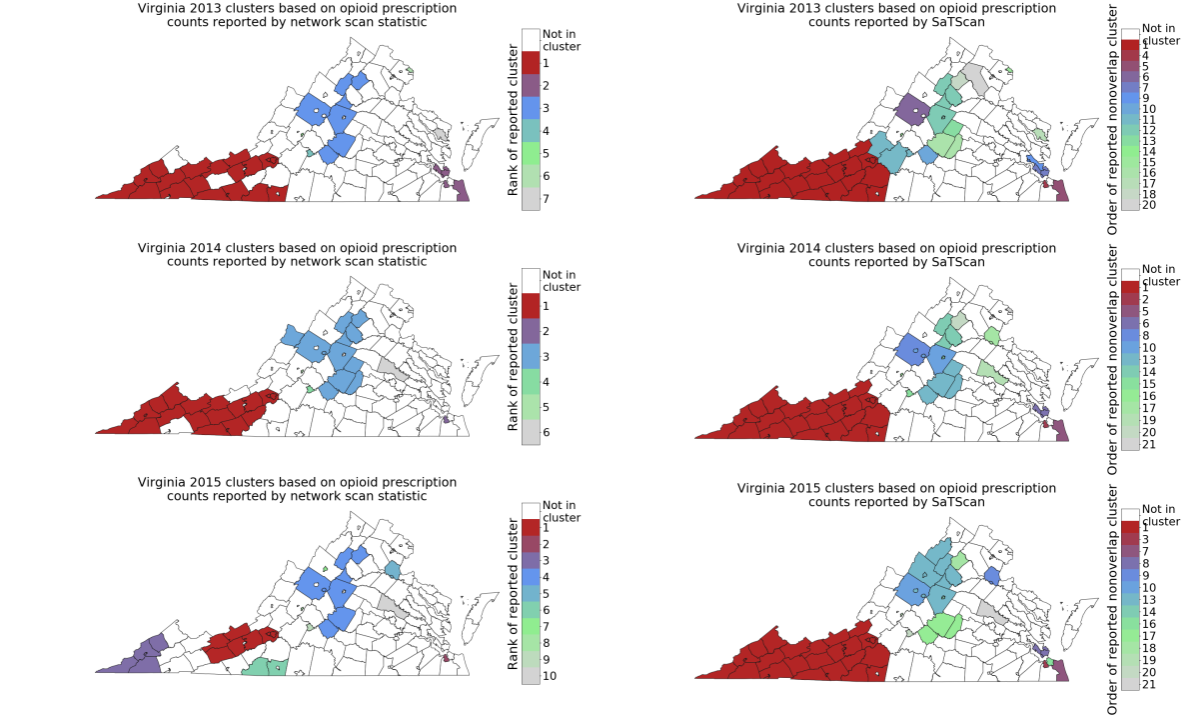
Clusters discovered in Virginia for 2013 (top), 2014 (middle), and 2015 (bottom), using network-based approach (left) and SaTScan (Martin Kulldorff, SaTScan; right) on opioid prescription counts.

Figure 2 shows the clusters with respect to opioid prescription counts for each year, using the network-based approach. The sizes and composition of these clusters are reported in the Multimedia Appendix 1. For 2013 and 2014, the clusters computed using SaTScan (Martin Kulldorff, SaTScan) cover a large part of the state. For 2014 and 2015, SaTScan (Martin Kulldorff, SaTScan) returned more clusters than the network-based approach. However, the likelihood scores of the clusters from SaTScan (Martin Kulldorff, SaTScan) were lower than those using network scan statistics, as the former only considers circular shapes.

We observed a similar layout in 2013 and 2014, as for beneficiary counts (Figure 1), the most significant cluster is in southwestern Virginia, with a few clusters in other parts. However, in 2015, the most significant cluster in the southwest part has shrunk and become concentrated in the western part. Furthermore, we find more significant clusters with respect to prescription counts. We also find the top clusters exhibit very similar characteristics with respect to different demographic properties, as reported above for the beneficiary counts.

#### Significant Temporal Changes Using the Expectation-Based Scan Statistic

**Figure 3:**
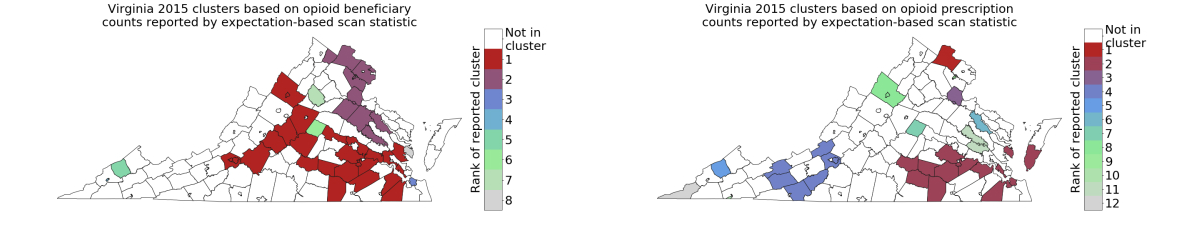
Clusters computed using the expectation-based scan statistic for 2015 using the county-level opioid beneficiary counts (left) and opioid prescription counts (right) in Virginia.

Figure 3 shows the significant clusters using the expectation-based scan statistics for 2015, using an average of counts from 2013 and 2014 for H_0_. We noted that these are quite different from the clusters computed using the Kulldorff statistics in Figure 1, especially for opioid beneficiary counts. In particular, there is no significant cluster in southwest Virginia in Figure 3, which is consistent with the limited change over time in Figure 1. Table 1 lists the size and composition of the top 2 clusters. As mentioned above, these are quite different from those reported by the network scan statistic in Figure 1 (left column); the most significant cluster is in central Virginia.

**Figure 4:**
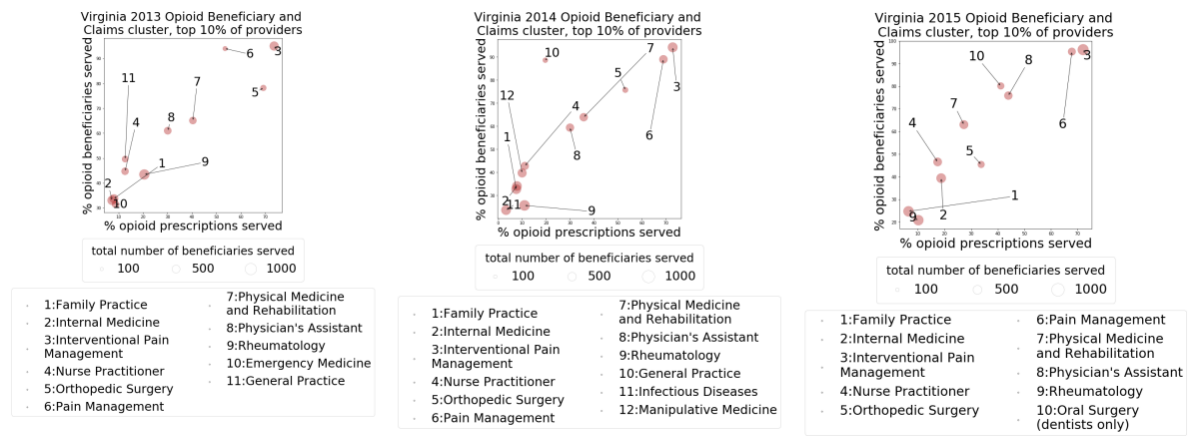
Scatter Plots for Virginia (VA) for years 2013, 2014, and 2015, showing the distribution of the provider specialties with respect to the mean of the percentage of opioid beneficiaries and prescriptions served by providers who are in the top 10 percentile of the providers in the anomalous clusters.

We then focused on the top 10 percentile of the top cluster for years 2013, 2014, and 2015. We selected providers who are in the top 10 percentile of the opioid beneficiary cluster and in the top 10 percentile of the top opioid prescription claims cluster. The scatter plot of Virginia in Figure 4 highlights the specialties of these top providers and plots their 4 attributes, that is, total beneficiaries, percentage of opioid claims, percentage of opioid beneficiaries, and medical specialties. The points that occur close together share common profiles.

### Characterizations of Demographics in Clusters

We now provide results from a regression-based analysis, which aims at characterizing all the counties that are a part of the anomalous clusters. Table 2 shows logistic regression–based results for Virginia for the opioid beneficiary data, and Table 3 shows a similar table for opioid prescription claims data. The coefficients represent the odds ratio. The detailed regression results for North Carolina and West Virginia are provided in the Multimedia Appendix 1.

The results show that a high percentage of African Americans in a county lowers the odds of the county being anomalous in terms of high opioid beneficiary count and high prescription count. The odds are fairly stable for opioid prescription claim counts across the 3 years at 0.94, but the odds change from 0.90 in 2013 to 0.96 in 2015 for opioid beneficiary count.

**Table 1:** Properties of the top 2 significant clusters (*P*<.05) in Virginia for opioid prescription counts and opioid beneficiary counts for the expectation-based scan statistic, for 2015, based on 2013 and 2014.

Beneficiary counts clusters	Prescription claim counts clusters
27 (Harrisonburg city, Rockingham, Albemarle, Nelson, Gloucester, Buckingham, Charles City, Goochland, Henrico, James City, Chesterfield, Isle of Wight, Suffolk city, Newport News city, Prince George, Petersburg city, Brunswick, Nottoway, Dinwiddie, Colonial Heights city, Sussex, Prince Edward, Roanoke city, Roanoke, Bedford, Salem city, and Amherst)	1 (Loudon)
13 (Loudoun, Prince William, Manassas city, Fairfax, Clarke, Falls Church city, Arlington, Fredericksburg city, Stafford, Spotsylvania, Caroline, Essex, and King William)	15 (Mathews, Chesterfield, Northampton, Suffolk city, Norfolk city, Newport News city, Portsmouth city, Prince George, Petersburg city, Brunswick, Nottoway, Dinwiddie, Surry, Prince Edward, and Lunenburg)

**Table 2:** Logistic regression results for Virginia opioid beneficiary data. The response variable takes value 1 if the county belongs to an anomalous cluster-based on the opioid beneficiary counts and it takes value 0 if it does not. Empty cells refer to cases when a variable was not selected in the year by AIC.

Variables	2013		2014		2015	
	Coefficient	*P* value	Coefficient	*P* value	Coefficient	*P* value
Intercept	0.32	.22	19.10	.16	0.47	.47
%African American	0.90	<.001^a^	0.93	.001^a^	0.96	.005^b^
%American Indian	0.13	.15	0.004	.01^b^	1.55	.20
%AccessToMedicaid	1.16	.003^b^	0.14	.009^b^	—	—
%AccessToMedicare	—	—	—	—	1.08	.03^c^
IncomePoverty <0.5	1.09	.13	—	—	1.09	.06^c^
%AccessToDirCare	—	—	0.83	.05^c^	0.89	.07^c^

^a^Significance code ≤.001.

^b^Significance code ≤.01.

^c^Significance code ≤.10.

**Table 3:** Logistic regression results for Virginia opioid prescription claims data. The response variable takes value 1 if the county belongs to an anomalous cluster based on the opioid prescription claim counts, and it takes value 0 if it does not. Empty cells refer to cases when a variable was not selected in the year by AIC.

Variables	2013		2014		2015	
	Coefficient	*P* value	Coefficient	*P* value	Coefficient	*P* value
Intercept	0.27	.12	0.34	.22	3.19	.38
%AfricanAmerican	0.94	.001^a^	0.92	<.001^a^	0.94	.002^b^
%AmericanIndian	0.13	.14	0.06	.06^c^	0.18	.17
%AccessToMedicaid	1.15	.001^a^	1.19	<.001^a^	1.15	.009^b^
%AccessToMedicare	—	—	—	—	0.88	.04^c^
NumHousingUnits	1.0	.12	—	—	—	—

^a^Significance code ≤.001.

^b^Significance code ≤.01.

^c^Significance code ≤.10.

Results also show that a higher number of people in the county with access to Medicaid increases the odds of the county being anomalous to about 1.16 in terms of high opioid beneficiary count and high prescription count in Virginia. This relationship is persistent across years.

Similar to Medicaid, access to Medicare also positively correlates with the odds of a county having a higher number of opioid beneficiaries in Virginia, but it lowers the odds of a county being anomalous in opioid prescription claim count. This relationship only holds in 2015; in 2013 and 2014, there is no relationship between access to Medicare and the 2 types of counts.

Income-to-poverty ratio <0.5 is positively correlated with the odds of higher opioid beneficiary counts in Virginia, in 2015. In rest of the years, it does not show any relationship. Individuals with income-to-poverty ratio <0.5 and those who receive Medicaid are extremely poor, and both these variables have a positive relationship with the opioid beneficiary count.

The number of housing units in a county is not a significant factor in impacting the odds of opioid beneficiary or opioid prescription claims count in that county in Virginia. A higher percentage of people with access to direct purchase of insurance plans decreases the odds of a county having high opioid beneficiary count. Between 2014 and 2015, these odds change from 0.83 to 0.89.

A similar regression analysis of the expectation-based clusters in 2015 did not find any significant demographic variables that were correlated with the odds of a county being anomalous for both types of counts in all 3 states.

## Discussion

### Principal Findings

Our analysis highlights several similarities and differences among the spatial clusters in Virginia, North Carolina, and West Virginia, with respect to county-level opioid prescription and beneficiary counts. Some of our key observations were the following: (1) in all 3 states, *the top cluster* with respect to beneficiary counts was fairly stable in 2014 and 2015, and it has shrunk from 2013; (2) the clusters discovered using the Kulldorff scan statistic are different from the expectation-based statistic, partly because of this reason; (3) the *top clusters* in these states have similarities in terms of demographic characteristics, such as racial features (predominantly white, except in North Carolina), income and employment levels (lower than statewide levels), number of housing units (significantly lower than statewide levels), and health care coverage (generally more people have public insurance, including Medicare and Medicaid, fewer have private insurance, and fewer people have employee paid plans).

The regression analysis, which considers all significantly anomalous clusters in the state, identifies factors that are common among counties that belong to these clusters. The regression results for Virginia show that the percentage of African Americans in each county are negatively correlated with the odds of that county being anomalous in both types of counts. As there is over 95% negative correlation between the percentage of whites in the county and percentage of African Americans in each county, these results also suggest that a higher percentage of whites is positively correlated with the odds of the county being anomalous. These results are consistent with the findings of the President’s commission report on opioid crisis [1], the CDC, and NSDUH [2,9], which show that the prevalence of opioid-use disorder (OUD) is the highest among whites (72.29%) and only 9.23% among blacks. Other factors overrepresented among those reporting OUDs are males (57.39%) and low-income individuals (<50,000, 67.12%) [9]. Finally, we observe that some of the predictors exhibit significant changes from 2013 to 2015 in Table 2. For instance, the variable *%AmericanIndian* is significant only in year 2014. The coefficient of *%AccessToMedicaid* is stable and significant across all years in Table 3, but in Table 2, the odds drop from 1.16 in 2013 to 0.14 in 2014. The reasons for this are not clear, and there might be other factors at play, which are not evident in the datasets used in this study.

In Virginia, there is a persistent correlation between a higher number of people in the county with access to Medicaid and the increase in the odds of the county being anomalous in both types of counts. As more people in a county depend on Medicaid services, the odds of having higher counts of opioid beneficiaries and prescriptions claim count go up. This may not be surprising as many of the government-run Medicaid programs have often come under fire for various types of waste, fraud, and abuse [31]. These are often because of improper payments to an ineligible beneficiary or an ineligible service or an ineligible provider. States have little incentive to dedicate their limited resources to track and recover these payments as states only get to keep their share of funding which is less than half. In 2017, US Department of Health and Human Services made $64 billion in improper payments [32].

The public health insurance programs may be more at risk of prescribing opioids because of lack of consistent policies toward *utilization management* such as *step-therapy* and *quantity limits* [8]. These utilization management rules require that the treatment start with a less risky drug that may be available over the counter and restrict the number of pills that can be prescribed at any given time. This is also consistent with the findings from previous research that low-income individuals are at a much higher risk of OUDs, as many of the Medicaid recipients are extremely poor [1,9].

Our results also show that network-based scan statistics [25] can give new insights into spatiotemporal hot spots of prescription opioids. The clusters we found were at a higher resolution than the circular clusters computed using SaTScan (Martin Kulldorff, SaTScan), and they have a higher likelihood score; thus, combining our approach with SaTScan (Martin Kulldorff, SaTScan) can be useful in practice. Finally, the Kulldorff and expectation-based scan statistics give different insights. In particular, the expectation-based approach can help identify clusters with significant change over time, which is not easy to determine from the Kulldorff clusters, which seem to be fairly stable in 2014 and 2015.

An analysis of the top 10 percentile of the top cluster in Virginia for years 2013, 2014, and 2015 shows some unusual patterns. We focus on the specialties of the providers who are in the top 10 percentile of the opioid beneficiary cluster and in the top 10 percentile of the top opioid claims cluster. The scatter plot of Virginia in Figure 4 shows that the points that occur close together share common profiles. For example, in years 2013 and 2014, physician assistants’ and physical medicine and rehabilitation specialists’ profiles were very similar. This seems unusual as these professions attend to very different sets of patients [33,34]. Physical medicine and rehabilitation specialists often treat patients with disabilities affecting the brain, spinal cord, nerves, bones, joints, ligaments, muscles, and tendons, whereas physician assistants have more comprehensive responsibilities, which include preventive health care, primary health care, and counseling of patients, etc. Similarly, in 2015, physician assistants and dental oral surgeons had a very similar profile, which is also unexpected as oral surgeons often deal with tooth extraction, implants, oral cancer, jaw surgery, etc—conditions that require significant pain management on the surgeon’s part [35].

Finally, a word cloud–based analysis (not shown here) found that a majority of the opioid beneficiaries in the most anomalous cluster are being served by physician assistants, nurse practitioners, and family practitioners. This holds true across the 3 years. We found this to be curious, as provider services in these specialties are often not focused on pain management, although they may be treating chronic pain [36,37]. High occurrences of these medical specialties in the top cluster may require further investigation by domain experts.

This research provides a generic methodology for identifying highly refined spatial hot spots that are likely to encounter prescription opioid misuse and overdose. This information can be used to monitor providers and their prescription behaviors in regions that are at a high risk of abuse. The anomalous regions can be targeted for better public health surveillance, access to treatment and recovery services, and availability and distribution of overdose-reversing drugs.

### Limitations

The Medicare Part D Prescription Drug data (*Methods/Datasets/CMS Data* section) has several limitations in the context of revealing hot spots of prescription opioid overdose. These data only comprise prescriptions paid by Medicare; only about 39.5 million people (a little over 10% of the total population) were covered in 2015 [38]. Furthermore, Medicare only covers a specific part of the population, namely people over 65 and those with certain disabilities. Finally, our analysis only considers counts of prescriptions and beneficiaries associated with opioids—the clusters we find are not necessarily hot spots of opioid overdose.

However, the demographics of people covered by Medicare are often associated with use of prescription opioids (though not necessarily their misuse); furthermore, about 40.6% of people reported obtaining prescription opioids from family or friends [1]. Therefore, we believe such an analysis using the CMS data is still valuable.

Another limitation of this study is that it does not account for spatial correlations of observations, which may exist at spatial boundaries and may impact the estimates of the standard errors.

### Conclusions

Though misuse of prescription opioids has been recognized as one of the significant factors driving the opioid overdose crisis, there has been limited understanding of the spatiotemporal characteristics of opioid prescription rates. All previous spatiotemporal analysis of metrics directly or indirectly related to opioid prescription rates and overdose has been restricted to a single state [4,13,14,22]. Our analysis using Medicare prescription rates is the first multistate spatiotemporal analysis of prescription rates for opioids. Even though this does not directly contain opioid overdose data, the clusters are likely to be relevant, as people represented in Medicare are associated with greater use of prescription opioids.

The regression analysis characterizes the demographics of the significant clusters associated with high opioid beneficiaries and prescription claims. Scan statistics is a useful technique for spatiotemporal analysis, but the network-based approach gives higher resolution clusters than SaTScan (Martin Kulldorff, SaTScan); the clusters we found have higher likelihood scores. Furthermore, the expectation-based statistic reveals different clusters than the Kulldorff scan statistic.

## Appendix

Multimedia Appendix 1 Supplementary appendix section to paper with studies on counties in North Carolina and West Virginia.

## Abbreviations

ACS: American Community Survey
AIC: Akaike Information Criterion
CDC: Centers for Disease Control and Prevention
CMS: Centers for Medicare & Medicaid Services
NSDUH: National Survey on Drug Use and Health
NSF: National Science Foundation
OUD: opioid-use disorder
PUF: Public Use File
