# Supplementary material for: Detection of Spatiotemporal Prescription Opioid Hot Spots With Network Scan Statistics: Multistate Analysis
Source: JMIR Public Health Surveill. 2019 Jun 17;5(2):e12110. doi: 10.2196/12110 (PMC6601258; doi:10.2196/12110)
Supplement: Multimedia Appendix 1 [file publichealth_v5i2e12110_app1.pdf]

Appendix

Significant Clusters in Virginia

Table 1. Counties in the significant clusters ($p < .05$) in Virginia computed using network scan statistics, with respect to the opioid beneficiary counts for each year. The cluster number in column 1 are in decreasing order of statistical significance.

Rank of cluster	Size of cluster and list of counties in the cluster for each year		
	2013	2014	2015
1	30 (Augusta, Nelson, Buckingham, Mecklenburg, Charlotte, Appomattox, Roanoke, Patrick, Henry, Pulaski, Franklin, Giles, Craig, Radford city, Salem city, Washington, Wise, Dickenson, Lee, Russell, Buchanan, Scott, Norton city, Smyth, Carroll, Bland, Bath, Rockbridge, Buena Vista city, Alleghany)	22 (Roanoke, Patrick, Henry, Pulaski, Franklin, Giles, Radford city, Salem city, Washington, Wise, Dickenson, Lee, Russell, Buchanan, Scott, Norton city, Grayson, Smyth, Carroll, Wythe, Bland, Galax city)	20 (Roanoke, Patrick, Henry, Pulaski, Franklin, Giles, Radford city, Salem city, Washington, Wise, Dickenson, Lee, Russell, Buchanan, Scott, Norton city, Smyth, Carroll, Wythe, Bland)
2	6 (Fauquier, Rappahannock, Shenandoah, Page, Madison, Greene)	4 (Virginia Beach city, Newport News city, Hampton city, Portsmouth city)	4 (Virginia Beach city, Newport News city, Hampton city, Portsmouth city)
3	1 (Hopewell city)	(Frederick, Rappahannock, Shenandoah, Page, Greene)	11 (Fauquier, Rappahannock, Page, Harrisonburg city, Rockingham, Augusta, Albemarle, Greene, Bath, Highland, Alleghany)
4	1 (Harrisonburg city)		1 (Hopewell city)
5			7 (Fredericksburg city, Stafford County, Caroline County, Essex County, Westmoreland County, Hanover County, King William County)

6			2 (Petersburg city, Colonial Heights city)
7			1 (Charles City County)

Summary Results for North Carolina

In North Carolina, we find one large cluster using network scan statistics in 2013 stretching along central North Carolina, which shrinks in 2014 and 2015, for the beneficiary counts data. The same central region of North Carolina is also a part of the largest cluster for the prescription counts data, and this cluster is expanding over the three years.

Significant clusters for North Carolina

We now discuss the results for North Carolina, following the same structure as Virginia in Section *Significant clusters for Virginia*. We omit some of the details of the clusters for brevity. Figure 1 shows the significant clusters for 2013-2015. We find one large cluster in 2013 stretching along central North Carolina, which shrinks in 2014 and 2015. A different second cluster also becomes significant in 2014 and 2015. The general demographic characteristics of these clusters are similar to those for Virginia. Some of the noticeable differences for North Carolina are: (1) The racial composition (predominantly white) seems to hold for 2013 and 2014. However, in 2015, the fraction of African American population is overrepresented in the top clusters; (2) Similarly, in 2014 and 2015, the top clusters have lower healthcare coverage of any type, compared to the state wide average; (3) The income to poverty levels and mean household income are both lower in 2014 and 2015. These results suggest changing trends over time, which might be important to consider for policy planning; (4) The patterns for health care coverage are similar to those in Virginia, except for direct care, which are lower for the top cluster (though not necessarily for the second cluster, as in 2015).

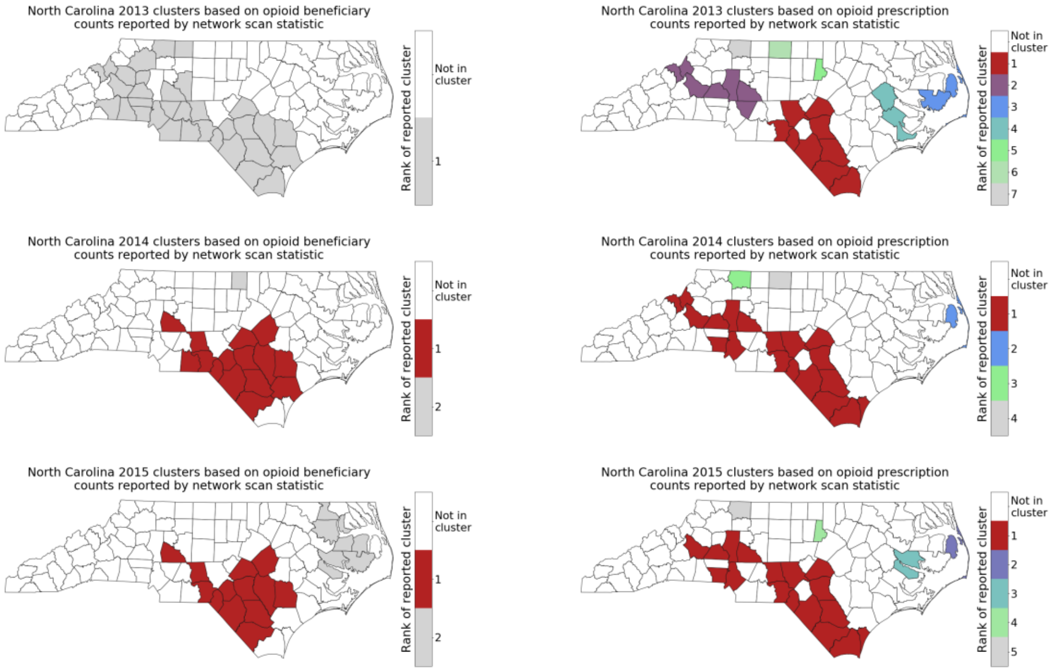

Figure 1. Clusters discovered in North Carolina for 2013 (top), 2014 (middle) and 2015 (bottom), using network-based approach on opioid beneficiary counts (left) and opioid prescription counts (right).

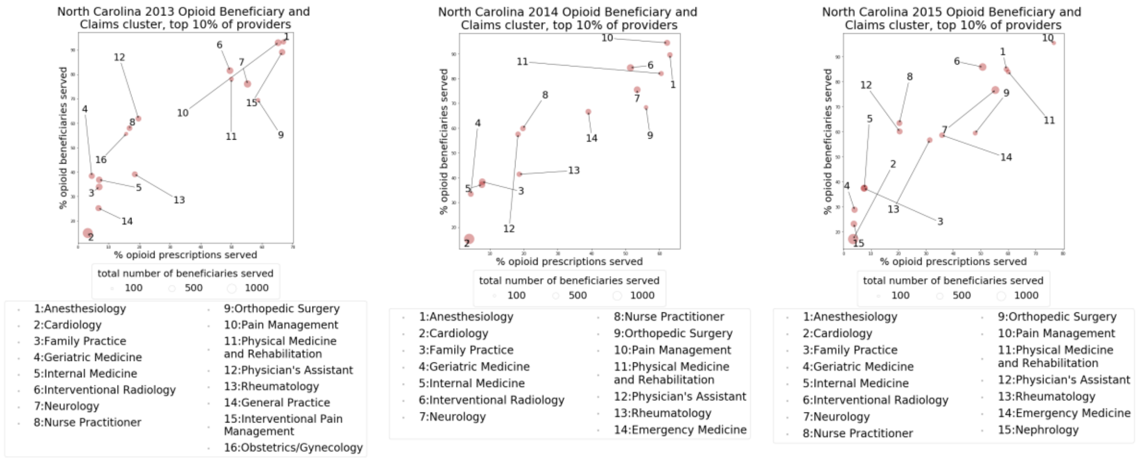

Figure 2. Scatter Plots for North Carolina for years 2013, 2014 and 2015, showing the distribution of the provider specialties with respect to the mean of the percentage of opioid beneficiaries and prescriptions served by providers who are in the top 10 percentile of the providers in the anomalous clusters.

The scatter plot in Figure 2 is for North Carolina providers who are in the top 10 percentile of the top cluster with respect to opioid claims and opioid beneficiaries, for years 2013, 2014 and 2015. Each year, the nurse practitioners and physician assistants appear to have very similar profiles. Even though they do not serve as high proportions of opioid claims and beneficiaries as surgeons and pain management specialists, their

significant presence in the top 10 percentile of the top cluster makes them unusual specialties.

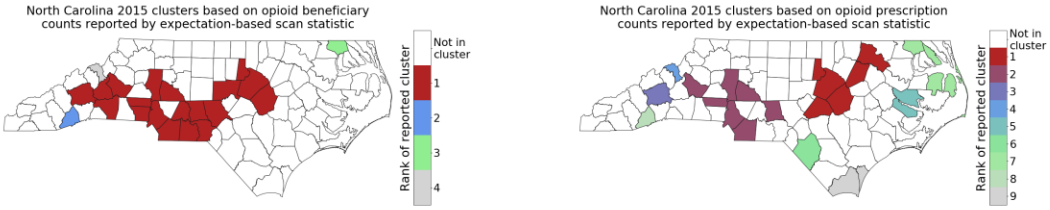

Figure 3. Clusters computed using the expectation-based scan statistic for 2015 using the county level opioid beneficiary counts (left) and opioid prescription counts (right) in North Carolina.

Summary Results for West Virginia

In West Virginia, the prescription counts cluster is fairly large and stable over the years but the beneficiary counts cluster is shrinking. Again, there is a lot of the spatial region in the southwestern part of West Virginia that is common across both types (beneficiary count and prescription count) of clusters, implying that there may be some systematic issues with healthcare services in these regions.

Significant clusters for West Virginia

Finally, we describe our results for West Virginia. Figure 4 shows the significant clusters for the network scan statistics. The top cluster is fairly stable between 2014 and 2015, after shrinking from 2013. The demographic characteristics are generally similar to those for Virginia and North Carolina. The patterns for healthcare coverage are closer to those for North Carolina.

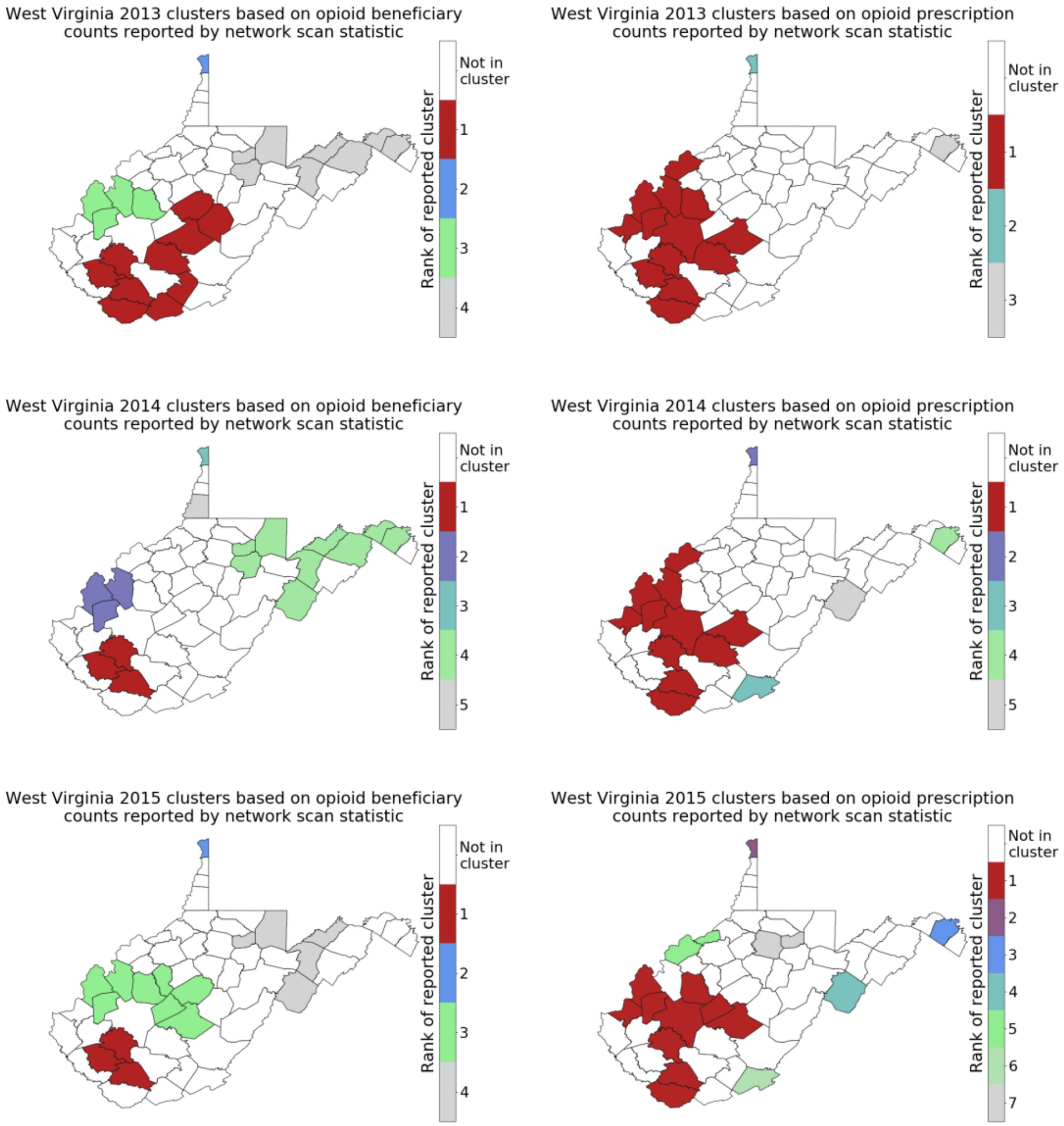

Figure 4. Clusters discovered in West Virginia for 2013 (top), 2014 (middle) and 2015 (bottom), using network-based approach on opioid beneficiary counts (left) and opioid prescription counts (right).

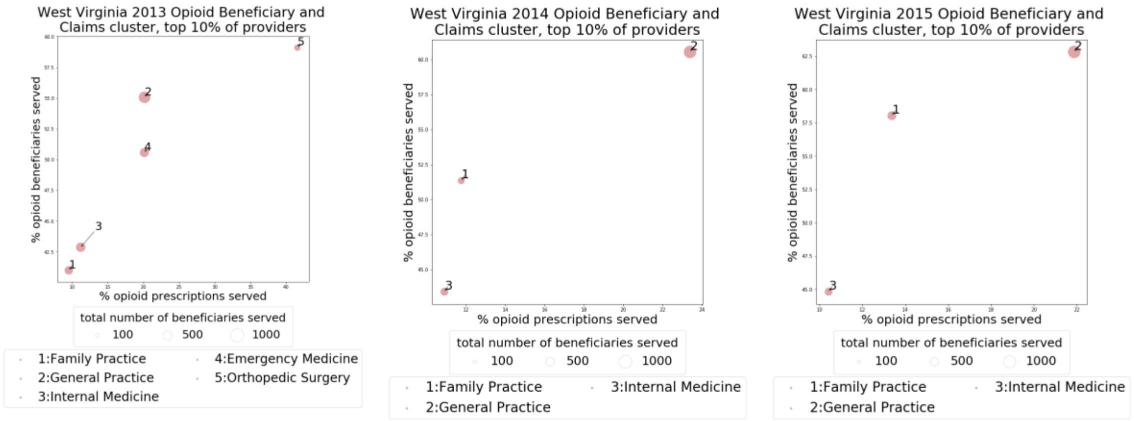

Figure 5. Scatter Plots for West Virginia for years 2013, 2014 and 2015, showing the distribution of the provider specialties with respect to the mean of the percentage of opioid beneficiaries and prescriptions served by providers who are in the top 10 percentile of the providers in the anomalous clusters.

The scatter plot of West Virginia in Figure 5 is for providers who are in the top 10 percentile of the top cluster with respect to opioid claims and opioid beneficiaries, for years 2013, 2014 and 2015. In West Virginia only a few specialties appear in the top 10 percentile, unlike North Carolina and Virginia. Here in all 3 years the general practitioners appear highly unusual as they serve a very high number of total beneficiaries as well as a high proportion of opioid beneficiaries and opioid claims.

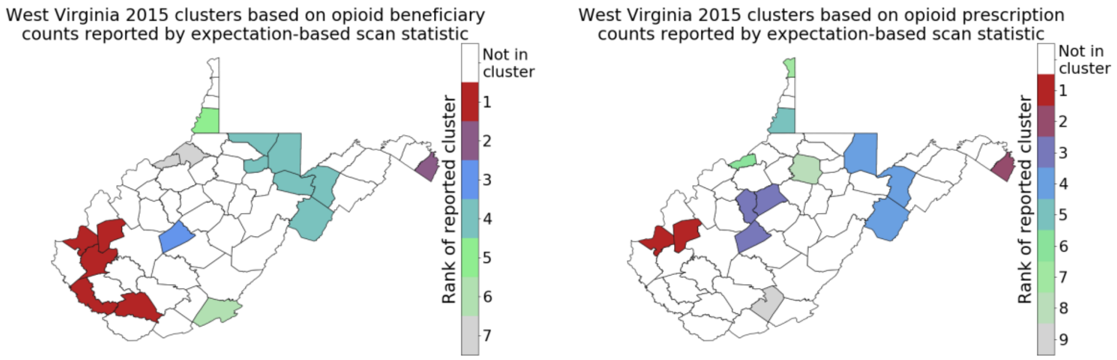

Figure 6. Clusters computed using the expectation-based scan statistic for 2015 using the county level opioid beneficiary counts (left) and opioid prescription counts (right) in West Virginia.

Table 3. Logistic regression results for North Carolina opioid-beneficiary data. The response variable takes value 1 if the county belongs to an anomalous cluster based on the opioid-beneficiary counts and it takes value 0 if it does not. Empty cells refer to cases when a variable was not selected in the year by AIC.

Variables	2013		2014		2015	
	Coefficient	P value	Coefficient	P value	Coefficient	P value
Intercept	18.54	.22	3394.8	.03[c]	0.008	.008[b]
%AfricanAmerican	0.89	<.001[a]	—	—	—	—
%AmericanIndian	0.92	.13	—	—	—	—
%AccessToMedicaid	1.29	.004[b]	0.14	.07[c]	1.42	<.001[a]
%AccessToMedicare	—	—	—	—	0.79	.01[b]
NumHousingUnits	1.00	.06[c]	—	—	—	—
%AccessToDirCare	0.62	<.001[a]	0.38	<.001[a]	—	—

[a]Significance code ≤ .001.

[b]Significance code ≤ .01.

[c]Significance code ≤ .10.

Table 4. Logistic regression results for North Carolina opioid-prescription claims data. The response variable takes value 1 if the county belongs to an anomalous cluster based on the opioid-prescription counts and it takes value 0 if it does not. Empty cells refer to cases when a variable was not selected in the year by AIC.

Variables	2013		2014		2015	
	Coefficient	P value	Coefficient	P value	Coefficient	P value
Intercept	—	—	0.02	.004[b]	0.05	<.001[a]
%AfricanAmerican	—	—	0.95	.03[c]	—	—
%AccessToMedicaid	—	—	1.17	.03[c]	—	—
IncomePoverty < 0.5	—	—	—	—	1.15	.11
NumHousingUnits	—	—	1.00	.014[c]	1.00	.03[c]

[a]Significance code ≤ .001.

[b]Significance code ≤ .01.

[c]Significance code ≤ .10.

Table 5. . Logistic regression results for West Virginia opioid-beneficiary data. The response variable takes value 1 if the county belongs to an anomalous cluster based on the opioid-beneficiary counts and it takes value 0 if it does not. Empty cells refer to cases when a variable was not selected in the year by AIC.

Variables	2013		2014		2015	
	Coefficient	P value	Coefficient	P value	Coefficient	P value
Intercept	7.95e+18	.03[c]	1.56e+12	.08[c]	1.06	.87
%AfricanAmerican	—	—	0.70	.07[c]	0.61	.04[c]
%Males	0.47	.04[c]	0.68	.16	—	—
%AccessToMedicaid	—	—	0.78	.02[c]	—	—
NumHousingUnits	1.00	.17	—	—	—	—
AccessToDirCare	0.66	.02[c]	0.70	.06[c]	—	—

[a]Significance code ≤ .001.

[b]Significance code ≤ .01.

[c]Significance code ≤ .10.

Table 6. Logistic regression results for West Virginia opioid-prescription claims data. The response variable takes value 1 if the county belongs to an anomalous cluster based on the opioid-prescription counts and it takes value 0 if it does not. Empty cells refer to cases when a variable was not selected in the year by AIC.

Variables	2013		2014		2015	
	Coefficient	P value	Coefficient	P value	Coefficient	P value
Intercept	0.80	.95	0.0004	.08[c]	0.26	.004[b]
%AccessToMedicaid	1.19	.14	0.80	.12	—	—
%AccessToMedicare	—	—	1.72	.02[c]	—	—
IncomePoverty < 0.5	0.70	.15	—	—	—	—
NumHousingUnits	1.00	.004[b]	1.00	.005[b]	1.00	.09[c]
AccessToDirCare	0.62	.02[c]	0.74	.06[c]	—	—

[a]Significance code ≤ .001.

[b]Significance code ≤ .01.

[c]Significance code ≤ .10.
